# Development, genetic mapping and QTL association of cotton *PHYA*, *PHYB*, and *HY5*-specific CAPS and dCAPS markers

**DOI:** 10.1186/s12863-016-0448-4

**Published:** 2016-10-24

**Authors:** Fakhriddin N. Kushanov, Alan E. Pepper, John Z. Yu, Zabardast T. Buriev, Shukhrat E. Shermatov, Sukumar Saha, Mauricio Ulloa, Johnie N. Jenkins, Abdusattor Abdukarimov, Ibrokhim Y. Abdurakhmonov

**Affiliations:** 1 Center of Genomics and Bioinformatics, Academy of Sciences of the Republic of Uzbekistan, University Street-2, Qibray region Tashkent District, 111215 Uzbekistan; 2 Department of Biology, Texas A&M University, Colleges Station, TX 77843 USA; 3 USDA-ARS, Southern Plains Agricultural Research Center, 2881 F&B Road, College Station, TX 77845 USA; 4 USDA-ARS, Crop Science Research Laboratory, Mississippi State, MS 39762 USA; 5 USDA-ARS, Plant Stress and Germplasm Development Research, 3810 4th Street, Lubbock, TX 79415 USA

**Keywords:** Cotton, Phytochromes, CAPS and dCAPS, Linkage mapping, Fiber QTLs

## Abstract

**Background:**

Among SNP markers that become increasingly valuable in molecular breeding of crop plants are the CAPS and dCAPS markers derived from the genes of interest. To date, the number of such gene-based markers is small in polyploid crop plants such as allotetraploid cotton that has A- and D-sub-genomes. The objective of this study was to develop and map new CAPS and dCAPS markers for cotton developmental-regulatory genes that are important in plant breeding programs.

**Results:**

*Gossypium hirsutum* and *G. barbadense*, are the two cultivated allotetraploid cotton species. These have distinct fiber quality and other agronomic traits. Using comparative sequence analysis of characterized GSTs of the *PHYA1*, *PHYB*, and *HY5* genes of *G. hirsutum* and *G. barbadense* one *PHYA1*-specific *Mbo* I/*Dpn* II CAPS, one *PHYB*-specific *Alu* I dCAPS, and one *HY5*-specific *Hinf* I dCAPS cotton markers were developed. These markers have successfully differentiated the two allotetraploid genomes (AD_1_ and AD_2_) when tested in parental genotypes of ‘Texas Marker-1’ (‘TM-1’), ‘Pima 3–79’ and their F_1_ hybrids. The genetic mapping and chromosome substitution line-based deletion analyses revealed that *PHYA1* gene is located in A-sub-genome chromosome 11, *PHYB* gene is in A-sub-genome chromosome 10, and *HY5* gene is in D-sub-genome chromosome 24, on the reference ‘TM-1’ x ‘Pima 3–79’ RIL genetic map. Further, it was found that genetic linkage map regions containing phytochrome and *HY5*-specific markers were associated with major fiber quality and flowering time traits in previously published QTL mapping studies.

**Conclusion:**

This study detailed the genome mapping of three cotton phytochrome genes with newly developed CAPS and dCAPS markers. The proximity of these loci to fiber quality and other cotton QTL was demonstrated in two A-subgenome and one D-subgenome chromosomes. These candidate gene markers will be valuable for marker-assisted selection (MAS) programs to rapidly introgress *G. barbadense* phytochromes and/or *HY5* gene (s) into *G. hirsutum* cotton genotypes or vice versa.

**Electronic supplementary material:**

The online version of this article (doi:10.1186/s12863-016-0448-4) contains supplementary material, which is available to authorized users.

## Background

Single nucleotide polymorphisms (SNPs) and small insertion/deletion (indel) polymorphisms are widely-used molecular marker systems in plants [1]. SNP markers have several advantages including their abundance and stability, as well as opportunity for high-throughput genotyping assays [2]. Because SNPs occur in both coding and noncoding regions, they can be used for genetic diversity assessment, molecular evolutionary studies, and genetic mapping for traits of interest in crop species. In particular, ‘candidate’ gene SNP polymorphisms are of great interest to reliably associate phenotypes with potentially causal polymorphisms in crops [1]; therefore, SNPs, in particular ‘candidate’ gene-based markers are valuable tools for association mapping and marker-assisted selection (MAS) [3].

A SNP can be detected and utilized through different methods that include, but are not limited to, enzymatic and chemical mismatch assays, allele-specific PCR (ASP), nucleotide-amplified polymorphisms (SNAP), ligase chain reaction, single stranded confirmation polymorphism analysis (SSCP), di-deoxy fingerprinting, cleaved amplified polymorphic sequences (CAPS) and derived-CAPS [2, 4–6], and genotyping by sequencing (GBS) using next generation sequencing technology [7]. Each method has particular advantages and disadvantages, and the use of particular SNP detection methods depends on many factors including prior expertise and the availability of the suitable platform and equipment [2].

One of the most widely used SNP genotyping systems is composed of the CAPS [4] and dCAPS [5, 8, 9] methods. CAPS are based on restriction enzyme site polymorphisms detected after amplification of a locus by PCR. When such restriction sites are not available within the SNP locus, restriction site can be created during PCR amplification by using primer design to introduce new nucleotides adjacent to the SNPs of interest, making a synthetic restriction site in the amplified product allele (dCAPS). CAPS and dCAPS markers are widely used because they are (1) usually based on a known gene, (2) easy to develop and genotype using PCR and agarose gel electrophoresis, (3) needing only small amount of starting DNA, (4) feasible in a typical molecular biology laboratory, and (5) easy to score in a co-dominant/dominant fashion. Among them, the most important advantage is the ‘candidate’ gene-based feature of genotyped CAPS/dCAPS polymorphisms that increases the power of genetic mapping and reliable marker utilization in breeding programs [3]. As with other SNP genotyping methods, the application of CAPS and dCAPS genotyping is complicated in complex polyploid genomes (such as cotton and wheat) due to the presence of both paralogous and homeologous gene copies. CAPS and dCAPS markers can detect polymorphisms between homeologous sub-genomes (inter-homeologous SNPs) within individuals, as well as orthologous SNPs between individual genotypes (known as genome-specific polymorphisms or GSPs). CAPS and dCAPS markers are effectively target the GSPs that differentiate polymorphisms from only a single sub-genome of allopolyploid species, providing the opportunity to analyze polyploids as diploid organisms [10, 11].

In cotton, a SNP marker framework is being developed that is based the analysis of candidate genes [12, 13], EST and transcriptome sequencing [14–18] and whole genome sequencing [19]. With the emergence and application of high-throughput next generation sequencing (NGS) technologies and GBS, a large number of SNPs were detected and made available for the cotton research and breeding [7, 19–21]. SNP markers were used to validate fine mapping QTL regions associated with important fiber traits [22, 23] and genetic male sterility [24] in cotton. However, cultivated cottons have a large and complex tetraplpoid genome with two partially homoeologous sub-genomes: the A-sub-genome consisting of chromosomes 1–13 and D-sub-genome consisting of chromosomes 14–26. To date, only a few examples have been shown for the utilization of CAPS and dCAPS-based SNP genotyping in cotton [13, 25] although the merits and importance of these markers were clearly described in early genetic mapping studies [26]. There is a special need for the development of genome-specific CAPS and dCAPS markers for important cotton genes in order to facilitate rapid MAS programs that can be easily utilized by cotton breeders with limited access to high-throughput, expansive genomic facilities.

Here, the cotton phytochrome gene family and its signal transduction factor sequences were targeted to develop genome-specific CAPS and dCAPS SNP marker sets using comparisons Upland cotton *Gossypium hirsutum* and *G. barbadense* genome. Phytochromes and their signal transduction factors are the particular targets because of their multiple effects in plant development, and their involvement in a wide range of genetic/biochemical pathways [27], yield potential and productivity [28–32], plant flowering and architecture [33], cotton fiber quality [34–37], salt tolerance [38, 39], regulation of nitrate reductase [40, 41], in cold/freezing and drought tolerance [42–44], and in fungal disease resistance [45]. Previously, the cotton phytochrome gene family and its signal transduction factor *HY5* were characterized, and their molecular evolution was studied by our group [35, 46], and cotton phytochromes were preliminarily associated with cotton fiber quality traits [35]. Recently, the biotechnology potential of phytochromes in the improvement of major fiber quality traits, early flowering and maturity, and increased cotton yield potential in a targeted RNA interference study was also reported by our team [37, 47].

The objective of this study was to develop and map cotton phytochrome (*PHYA1* and *PHYB*) and *HY5*-specific CAPS and dCAPS markers using GSP sites that are polymorphic between *G. hirsutum* and *G. barbadense*. Further, these markers were validated and integrated into a reference genetic map of cotton, constructed by Yu et al. [48, 49], and the chromosomal assignments of CAPS and dCAPS markers were verified using chromosome substitution (CS-B) lines [50–52]. Further, we explored the association of these novel CAPS and dCAPS markers with cotton fiber traits that may be useful for MAS programs.

## Results and discussions

### Gene-specific CAPS and dCAPS marker development

Previously, one *PHYA1* gene specific *Bbv* I CAPS marker targeting the 213 bp hinge region of cotton *PHYA1* genes and detecting a G to A transition, was developed and validated in an interspecific mapping population that was segregating for fiber length [35]. The *G. barbadense* allele of the D-genome specific *PHYA1* locus was co-dominantly digested by BbvI into the ~113 and 100 bp products, while *G. hirsutum* allele remained undigested.

In this study, to obtain better exploitation phytocrome genes in our breeding programs, the flanking upstream and downstream regions of previously characterized GSTs [35, 36] were sequenced and additional CAPS and dCAPS markers using commonly available restriction enzymes were developed. Upon sequencing upstream and downstream regions of cotton *PHYA* and *HY5* genes, 2.2 kb long GSTs were obtained covering a part of first exons, second exons, and a part of third exon as well as the first and second introns of the cotton *PHYA* genes. The first, second and a part of third exon as well as first and second intron sequences for cotton *HY5* genes (data not shown) also were cloned and sequenced, which then were used to develop GSP-specific CAPS and dCAPS markers. The 2.1 kb cotton *PHYB* GSTs of cotton corresponding to the part of first exon (covering the hinge region), first intron and part of the second exon of *PHYB* genes (*PHYB1* and *PHYB2*) were already characterized [46], and these GSTs were searched to find suitable GSPs for marker development.

**Fig. 1:**
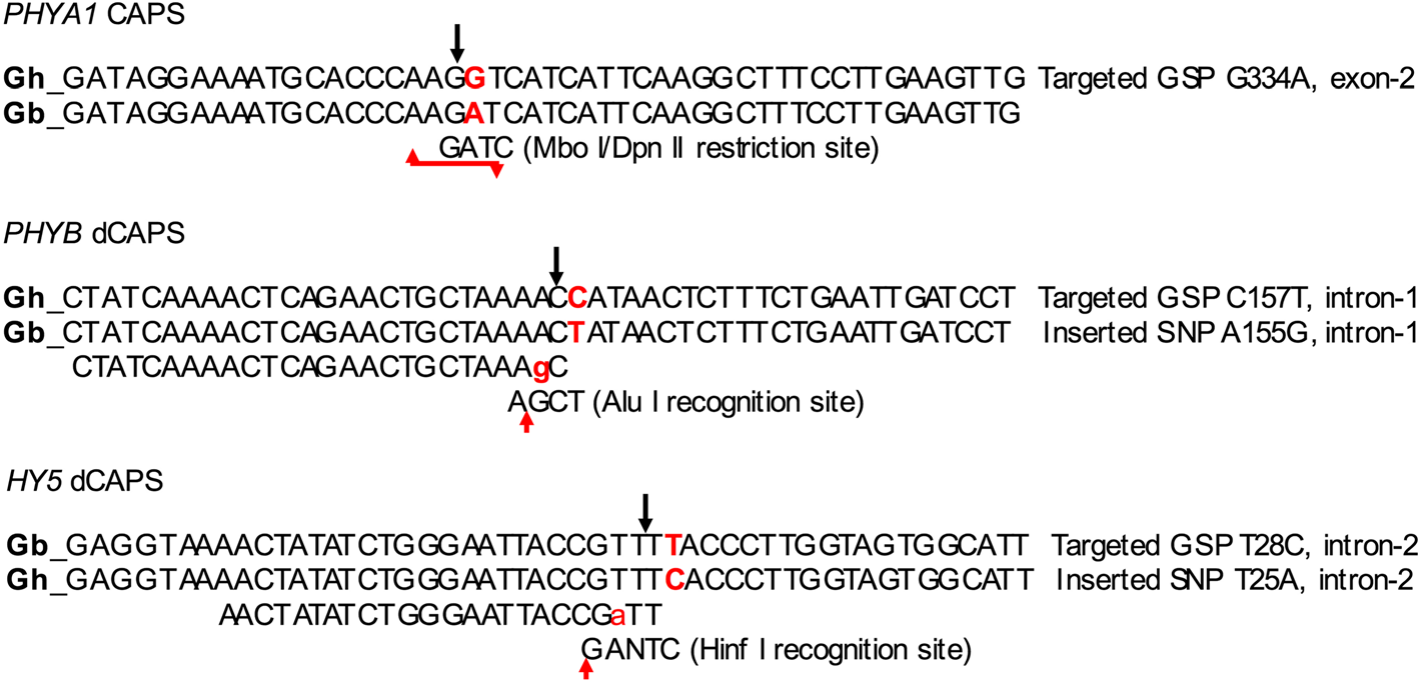
Targeted polymorphisms and restriction sites in phytochrome and *HY5*-specific CAPS and dCAPS markers. Gh - *G. hirsutum*, Gb - *G. barbadense*

**Fig. 2:**
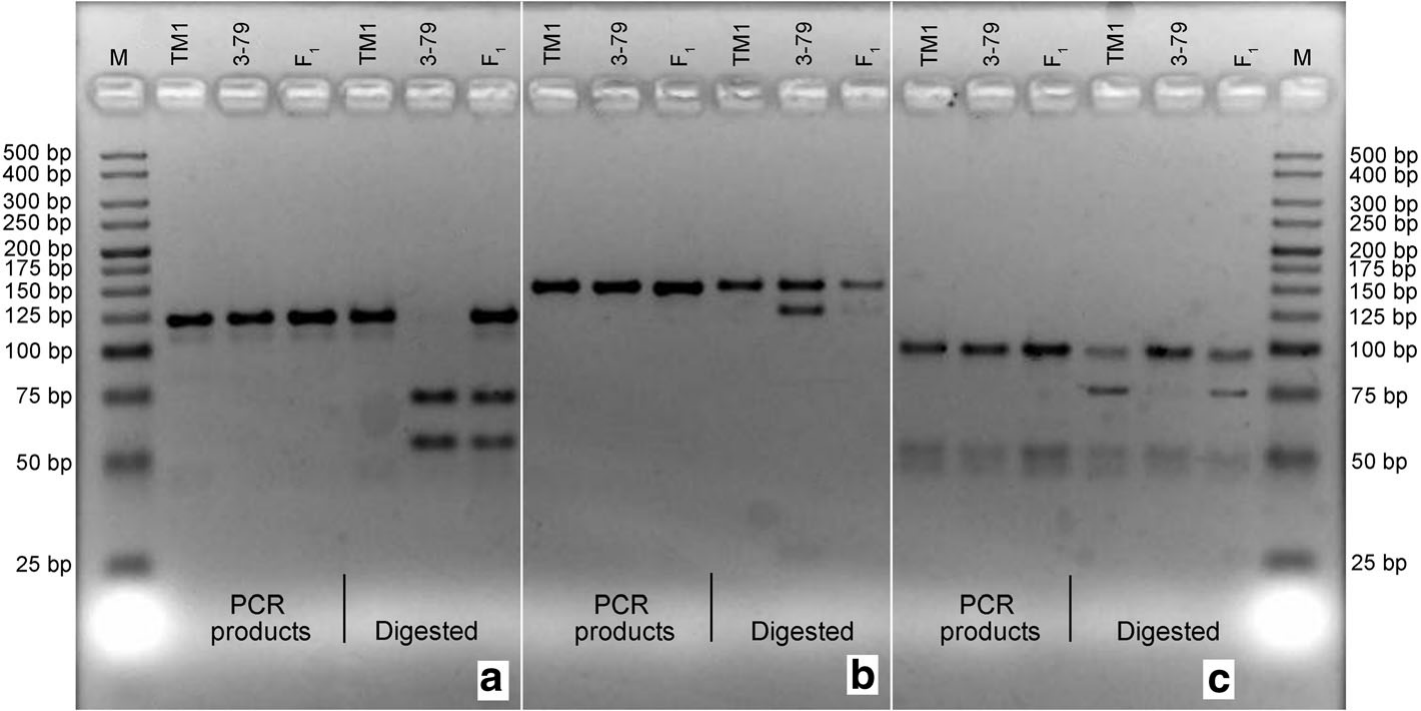
Agarose gel electrophoresis for undisgested and digested CAPS and dCAPS marker products: **a** *PHYA1* CAPS, **b** *PHYB* dCAPS, and **c** G.h.*HY5* dCAPS. (M) – Molecular-weight size marker of 25 bp ladder, ‘TM-1’ and ‘Pima 3–79’ – parents, F_1_ – first-generation hybrid. Note: in (**b**) and (**c**) there are 27 and 24 bp digestion products, migrating in a primer pair zone; therefore, hard to be relaibly detected

Using comparative sequence analysis of characterized GSTs *PHYA1*, *PHYB*, and *HY5* genes of *G. hirsutum* and *G. barbadense*, a total of 10 pairs of CAPS and dCAPS primer pairs were designed (not shown). Out of these 10 primer pairs, one cotton *PHYA1*-specific CAPS (with Mbo I/Dpn II endonuclease digestion sites), one *PHYB*-specific dCAPS (with Alu I restriction site), and one *HY5*-specific (with Hinf I restriction site) dCAPS primer pairs successfully differentiated between A- and D-sub-genomes when tested in parental genotypes of ‘TM-1’ [(AD)_1_], ‘Pima-3–79’ [(AD)_2_], and interspecific F_1_ hybrids (Table 1; Figs. 1 and 2a-c).

**Table 1:** Cotton phytochrome and *HY5*-specific CAPS and dCAPS markers

#	CAPS markers	Primer sequences (5’–3’)	PCR products (bp)	Restriction enzyme	Restriction products (bp)	
					*G. hirsutum* (‘TM-1’)	*G. barbadense* (‘Pima 3-79’)
1.	PHYA1_CAPS	F-5’TGCAAAGCAGGAACTTGGCA R-5’CATCCATTTGATAGTCCTTCCAC 3’	122	*Mbo* I *Dpn* II	51/71	122
2.	PHYB_dCAPS	F-5’CAACCTCAAAATCTGATGAAGTAAAC3’ R-5’CTATCAAAACTCAGAACTGCTAAAGC3’	149	*Alu* I	149	125/149
3.	GhHY5-2_dCAPS	F-5’AACTATATCTGGGAATTACCGATT3’ R-5’GTTTCGCAACAACCTCTTTTCA3’	97	*Hinf* I	27/70	97

In particular, a *PHYA1* CAPS marker was developed for D-genome derived *PHYA1* in tetraploid cottons, in which *PHYA1* CAPS primer pairs specifically amplified and differentiated from *PHYA2* locus. *PHYA1* CAPS primer pairs amplified one 122-bp *PHYA1* fragment from *Gossypium* genome, corresponding to a portion 743-bp second exon of the cotton *PHYA1* genes. This exon at the position of 334 had G to A transition mutation in *G. barbadense* that created GATC recognition site (versus GGTC in *G. hirsutum*). This G334A polymorphic site was recognized and digested by *Mbo* I/*Dpn* II endonuclease (Fig. 1) resulting in digestion of the *G. barbadense PHYA1* amplicon into 71- and 51-bp fragments. In contrast, *G. hirsutum* amplicons remained undigested, giving an opportunity to clearly differentiate *G. hirsutum* amplicon(s) from *G. barbadense* allele(s) in a co-dominant fashion (Fig. 2a).

Restriction enzyme recognition site polymorphisms targeted region of cotton *PHYB* genes were not found targeted region of cotton *PHYB* genes, although GSPs between *G. hirsutum* and *G. barbadense* were present. When an additional nucleotide (C157T) was incorporated nearby one of the existing GSP (A155G) of 388-bp first intron of *G. barbadense* using dCAPS primer mismatch approach, resulting amplicon had an AGCT *Alu* I recognition site in *G. barbadense* allele (Fig. 1). Thus, the *PHYB* dCAPS primer pair (Table 1) amplified 149-bp *PHYB* PCR product from both cotton species. When digested, *G. barbadense* allele yielded both the149-bp (undigested) fragment, as well as 125- and 24-bp digested bands, while *G. hirsutum* amplicon(s) showed no digestion (Fig. 2b; the 24-bp fragment migrates along with primers and primer-derived artifacts, and was not distinguished in the agarose gel). Therefore, *PHYB* dCAPS marker could only be scored as a dominant marker and heterozygotes could not be distinguished. It is noteworthy to mention here that one additional *PHYB* marker, designated PHYBdCAPS-2 with a *Hpa* I restriction polymorphism (refer to the Additional file 1: Figure S1a) was also designed. This dCAPS marker amplified 180-bp product from both *G. hirsutum* and *G. barbadense* genotypes (Fig. 5b). *Hpa* I digestion yielded 36- and 144-bp restricted and 180-bp unrestricted bands in both genotypes. In that, 144-bp band was more intensive in *G. hirsutum* and the unrestricted 180 bp band was more intensive in *G. barbadense* genotypes while 36-bp band was not visible to detect in agarose gel. Heterozygotes showed both intensive bands of 180- and 144-bp. This marker information is not included in main part of this paper because of complexity and a need for ‘band-intensity-based’ genotyping of restricted fragments (see Additional file 1: Figure S1a) that may generate inconsistent results by others when genotyped manually.

Further, using dCAPS approach, one *HY5*-specific dCAPS marker (*Hin*f I restriction site) was designed, targeting a T to C transition within the 90-bp second intron of *HY5* genes (Fig. 1). This dCAPS marker clearly differentiated *G. hirsutum* and *G. barbadense HY5* gene alleles (Fig. 2c) in dominant marker fashion, where *G. barbadense HY5* amplicon remained undigested (about 97 bp), whereas *G. hirsutum* amplicons were separated into 97-bp (undigested) as well as 70- and 27-bp digested fragments (where 27-bp fragment migrates on primer pair zone and not distinguished in agarose gel).

### Linkage mapping and QTL association analyses

Previously, the *PHYA1* CAPS marker specific to hinge region and recognized by *Bbv* I/*Bse* XI was amplified in fiber length segregating cotton population, an interspecific cross between ‘Pima S-7’ (*G. barbadense*) and ‘Tamcot SP37’ (*G. hirsutum*) consisting of 96 F_2_ individuals. Amplified products were digested with *Bbv*I endonuclease, and polymorphic bands were scored as co-dominant fashion. QTL-mapping of *Bbv* I CAPS marker polymorphism in a cotton fiber length segregating population revealed that the *PHYA1* locus is significantly linked to fiber length with LOD score of 4.27 and *p*-value of 0.00001 and explained about 6 % phenotypic variation [35]. This QTL association gave the preliminary molecular insights that phytochrome genes, and the *PHYA1* gene in particular, could be important in the fiber elongation process in cotton [35, 37].

**Fig. 3:**
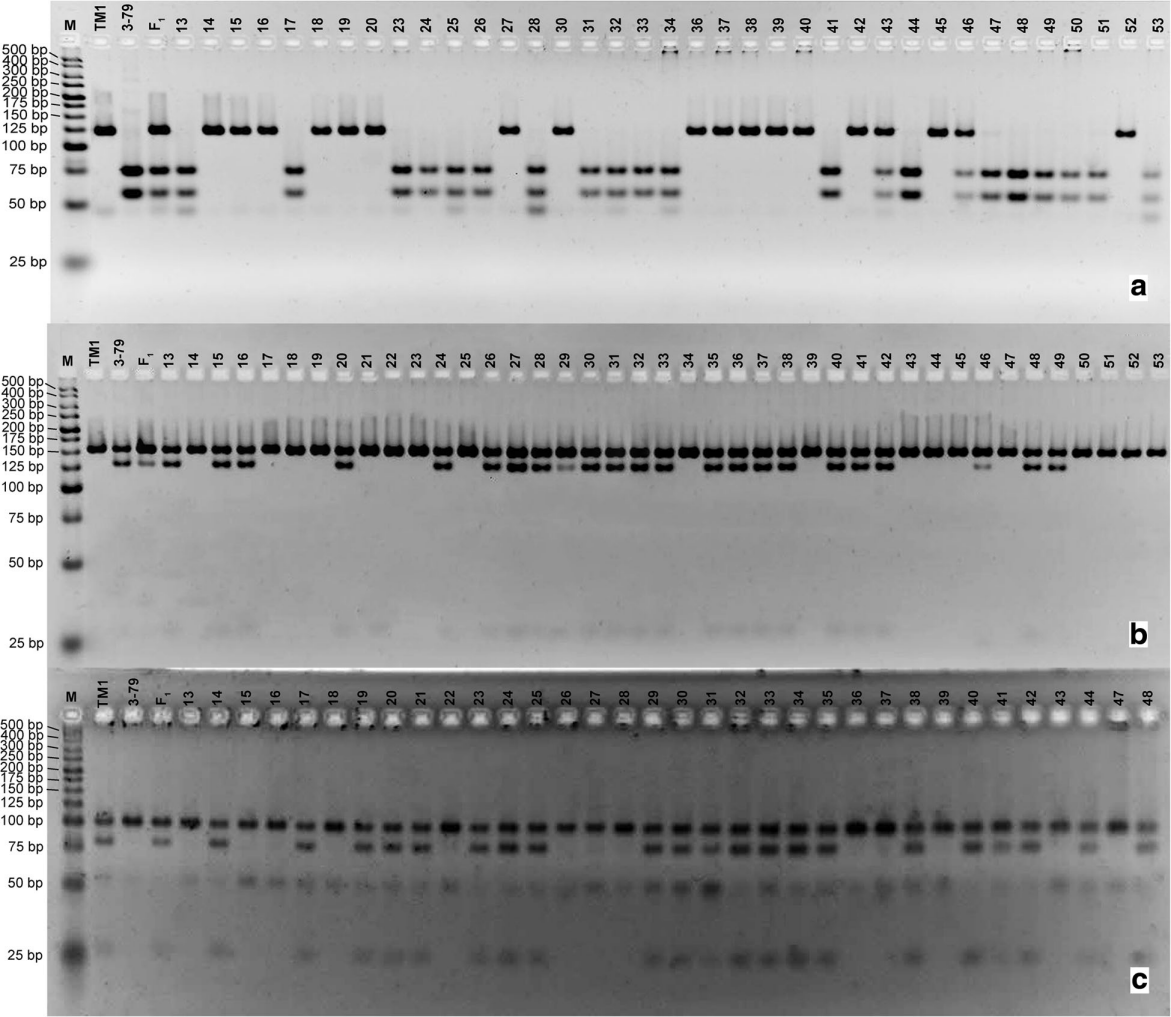
The examples of phytochrome and *HY5*-specific CAPS and dCAPS markers, segregating among ‘TM-1’ x ‘Pima 3–79’ RIL lines. **a** *PHYA1* CAPS, **b** *PHYB* dCAPS, and **c** Gh_*HY5* dCAPS. (M) – Molecular-weight size marker of 25 bp ladder, ‘TM-1’ and ‘Pima 3–79’ – parents, F_1_ – first-generation hybrid, 13–53 – RIL individuals

**Fig. 4:**
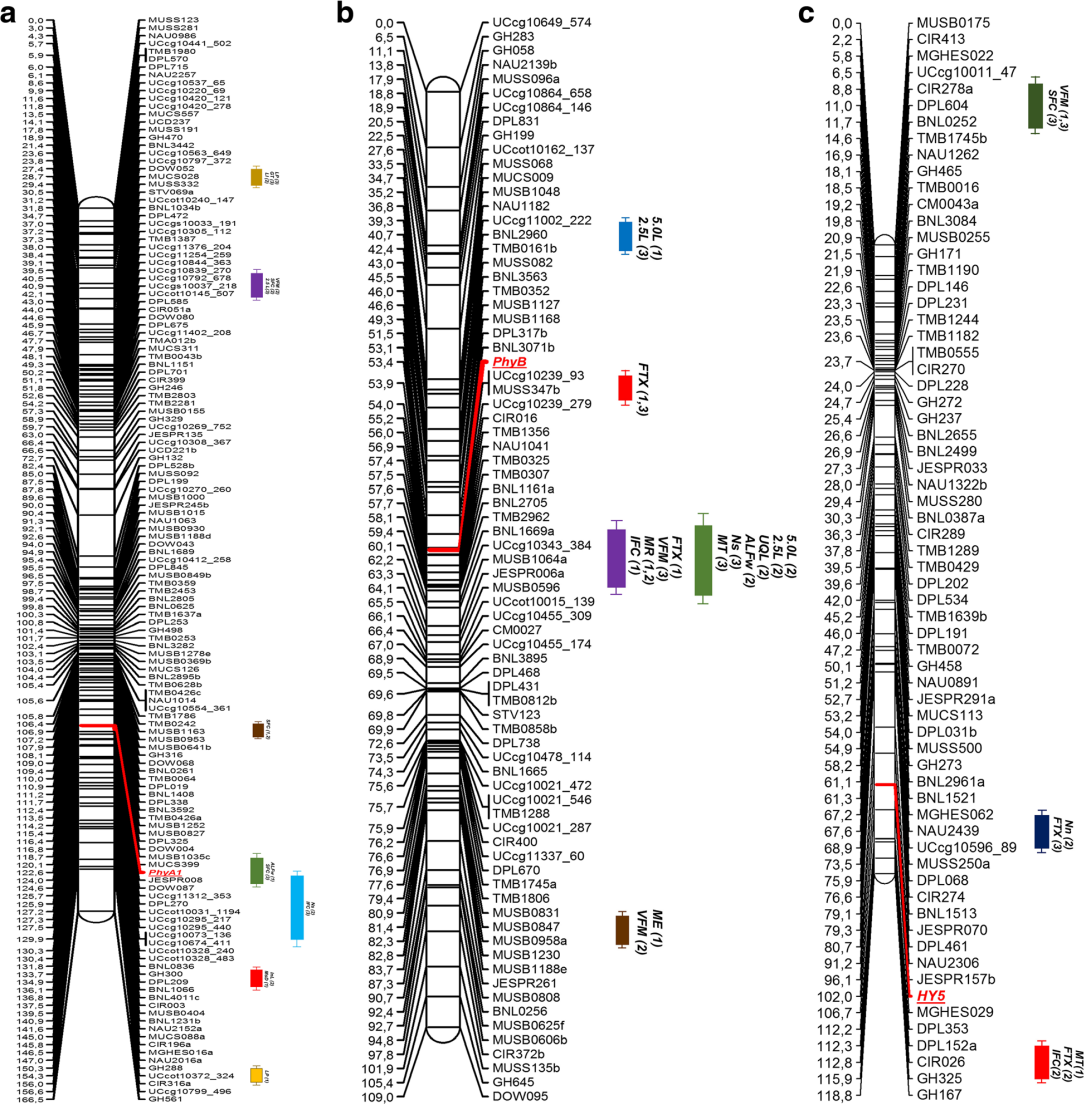
Genetic linkage maps with integration of phytochrome and *HY5*-specific CAPS and dCAPS markers. **a** *PHYA1* CAPS, A-subgenome chromosome 11, **b** *PHYB* dCAPS, A-subgenome chromosome 10, and **c** Gh_*HY5* dCAPS, D-subgenome chromosome 24. QTL designations on the map are follows as: InL - internode length; MsD - main stem diameter; LP - lint percent; GT - gin turnout; SI - seed index; LI - lint index; Ns - nep size; Nn - number of neps; SCN - number of seed coats; UQL - upper quartile of fiber length by weight; SFC - short fiber content by weight g; ALFw - average length of all fiber by weight; 5.0 L - fiber span length; 2.5 L - fiber span length; VFM – visible foreign matter in percentage; FTX - fiber fineness; IFC - immature fiber content by weight g; MR - maturity ratio; MT - mean tenacity; and ME - mean elongation

To study the possible genetic associations of phytochrome and *HY5*-specific CAPS and dCAPS markers with a suite of multi-environmentally evaluated complex traits (including all major fiber traits), these markers were incorporated into the reference genetic linkage map of cotton constructed using a large number SSR and SNP markers [48, 49]. Toward this goal, our candidate gene-specific CAPS and dCAPS markers were genotyped across all 186 RIL lines (Fig. 3a-c; Additional file 1: Figure S1a) from an interspecific cross between ‘TM-1’ and ‘Pima 3–79’ [48, 49]. Based on these data, the *PHYA1* CAPS marker was assigned into linkage group of A-sub-genome chromosome 11 (Fig. 4a). The *PHYB* dCAPS marker (Fig. 4b) and the PHYBdCAPS-2 marker (genotyped using band-intensity level) were assigned to chromosome 10 in the A-sub-genome, in a very close proximity to each other (Additional file 1: Figure S1b). The *HY5* dCAPS marker was assigned to linkage group 24 in the D-sub-genome [48, 49] (Fig. 4c). When the gene-based CAPS markers were placed on these three chromosomes, the mapping accuracy of individual CAPS marker positions was tested. There was no interspecific segregation distortion that would otherwise affect these CAPS loci.

Detailed study of flanking markers and QTLs associated with regions nearby our candidate gene markers revealed that *PHYA1* CAPS marker mapped between two SSR markers JESPER008 and MUCS399 at 3.925 cM distance (Fig. 4a). This region and flanking SSR markers were associated with such important fiber traits as short fiber content by weight (SFC) and an average length of all fibers by weight (ALFw) [48, 49]. In another independent QTL mapping study using testcross mating-design mapping population, Yu et al. [53] found association of one of the flanking markers linked to *PHYA1* CAPS, MUCS399, with micronaire (MC) and lint yield (LY). These results indirectly associate the *PHYA1* CAPS marker with these fiber quality traits and validate our previous findings on association of *PHYA1 Bbv* I/*Bse* XI CAPS with the fiber length trait [35]. Moreover, the results of our targeted RNAi study for *PHYA1* gene(s), improved fiber length and other key fiber quality traits, including short fiber content, microniare, strength, and uniformity [36, 37, 47], further support *PHYA1* CAPS marker associations with the related fiber QTLs discussed here.

Similarly, the *PHYB* dCAPS marker, on A-sub-genome chromosome 10, flanked by BNL3071 and Uccg10239_93/MUSS347b markers at 0.766 cM distance (Fig. 4b, Additional file 1: Figure S1b) were reported to be associated with fiber fineness (FTX) by Yu et al. [48, 49]. In another independent QTL mapping effort, Guo et al. [54] reported that BNL3071 marker, also tightly linked with *PHYB* dCAPS marker, was associated with the node of first fruiting branch (NFB) in a mapping population (F_2:5_) from the cross between T1107 and T1354, a day-neutral cultivar Deltapine 61 and photoperiodic *G. hirsutum* accessions, respectively. These findings further suggest the importance of phytochrome and light signal transduction for both fiber development and for flowering time/earliness in cotton.

The *HY5* dCAPS marker was flanked by markers JESPR157b and MGHES029 at 10.617 cM distance in the D-sub-genome chromosome 24 (Fig. 4c) [48, 49]. In an independent QTL mapping study mentioned-above, Yu et al. [53] reported the association of these two flanking markers linked to *HY5 Hin*fI CAPS, JESPR157b and MGHES029, with fiber uniformity (FU). A comprehensive meta-QTL analysis conducted by Said et al. [55] associated JESPR157a and CIR026 markers at 3.51 cM distance with a micronaire hotspot (‘c24-Micronaire-Hotspot-15’) including 4 QTLs, (Fig. 4c) [48, 49]. Yu et al. [48, 49] at the same time also associated this distal region of D-sub-genome chromosome 24 around CIR026 with immature fiber content (IFC), fiber fineness (FTX), and mean tenacity (MT). Additionally, Wang et al. [56] associated DPL461 marker with fiber elongation (FE) trait in a mapping population derived from an interspecific cross between *G. hirsutum* and *G. darwinii* Watt. The DPL461 is located at 21.255 cM distance to *HY5* dCAPS marker in D-sub-genome chromosome 24 (Fig. 4c) [49]. All these observations suggest the potential role of *HY5* genes in cotton fiber quality regulation.

### Verification of chromosomal locations using CS-B lines

**Fig. 5:**
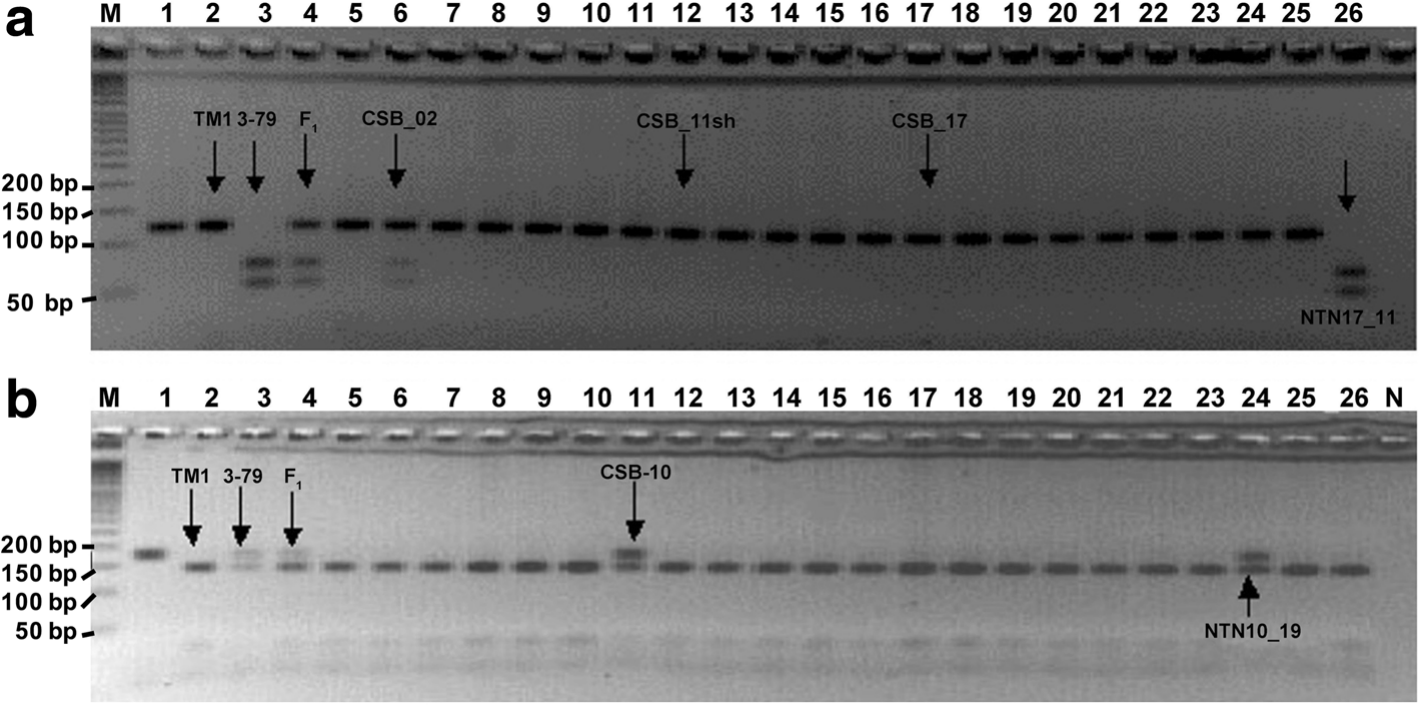
Chromosomal localization of cotton **a** *PHYA1* (*PHYA1* Alu I CAPS) and **b** *PHYB* (*PHYB* Hpa I dCAPS-2) genes using dCAPS markers on chromosome substitution lines (CS-B) and translocations (NTN). M-50 bp DNA ladder; 1-undigested PCR product; 2-‘TM-1’; 3- ‘Pima 3–79’; 4-F1; 5-CS-B_01; 6-CS-B_02; 7-CS-B_04; 8-CS-B_5sh; 9-CS-B_06; 10-CS-B_07; 11-CS-B_10; 12-CS-B_11sh; 13-CS-B_12sh;14-CS-B_14sh; 15-CS-B_15sh; 16-CS-B_16; 17-CS-B_17; 18-CS-B_18; 19-CS-B_22sh; 20-CS-B_22lo; 21-CS-B_25; 22-CS-B_26lo; 23-NTN_04-15; 24-NTN_10-19; 25-NTN_16-15; and 26-NTN_17-11

In addition, deletion analysis of dCAPS markers reconfirmed the chromosomal localization of cotton *PHYA1* and *PHYB* genes via linkage mapping analysis (Figs. 4 and 5). In both analyses, *PHYB*dCAPS-2 marker was assigned to an A-sub-genome chromosome 10 (Fig. 5b; Additional file 1: Figure S1b), with detailed linkage information of this marker with other ordered markers in relation to adjacent cotton QTLs (Fig. 4b) [48, 49]. Chromosomal localization of cotton *PHYA1* genes using cytogenetic stocks revealed that chromosomes 2 (not confirmed by mapping results) and 11 may bear *PHYA1* as the CS-B02 and NTN17_11 stocks showed the Pima specific band. However, neither CS-B17 (full) nor CS-B11 short arm (only available stock) had Pima specific allele; therefore, considering linkage mapping results it is likely that *PHYA1* is located on long arm of chromosome 11 (Fig. 5a).

## Conclusions

This study reports the genome mapping of three cotton phytochrome genes with newly developed CAPS and dCAPS markers. The proximity of these loci to fiber quality and other cotton traits was demonstrated in two A-sub-genome, and one D-sub-genome chromosomes. ‘Candidate’ gene specific CAPS and dCAPS markers developed for important plant genes such as *PHYA1*, *PHYB*, and *HY5* of cotton will be useful for cotton breeding programs worldwide for precise, targeted introgression important fiber and flowering traits from *G. barbadense* into *G. hirsutum* cultivars or vice versa. Further, flanking SSR markers closely linked with these CAPS and dCAPS markers, identified herein, and ready exploitation of these CAPS and dCAPS markers by breeders would further enhance the efficiency of MAS programs and foster the development of improved cotton cultivars.

## Methods

### Plant materials

The ‘Texas Marker-1’ (‘TM-1’, *G. hirsutum* L.), ‘Pima 3–79’ (*G. barbadense* L.) and 186 recombinant inbred lines (RILs) derived from an interspecific cross between ‘TM-1’ and ‘Pima 3–79’ were used in this study [48, 49]. These cotton genotypes were obtained from the USDA-ARS Cotton Germplasm Unit, College Station, Texas, USA. The cytogenetic stocks and CS-B line collection of tetraploid cotton [50–52] were used for the verification of chromosomal localizations of CAPS and dCAPS markers. The cytogenetic stocks and CS-B lines were kindly provided by Prof. D.M. Stelly, Texas A&M University, College Station, Texas, USA, and USDA-ARS partner laboratory, Starkville, Mississippi, USA through USDA-Uzbekistan cotton germplasm exchange program.

### DNA extraction and sequencing

Genomic DNAs were isolated from young leaves using the cetyltrimethylammonium bromide (CTAB) method [57]. The characterization, cloning, and sequencing of cotton phytochromes and *HY5* genes were performed as described by Abdurakhmonov [35] and Abdurakhmonov et al. [46].

### CAPS and dCAPS marker development

To develop phytochrome gene-specific CAPS and dCAPS markers, a PCR-walking experiment was designed to sequence upstream and downstream of previously sequenced hinge region [35, 46] of *PHYA* genes of *Gossypium* species. We successfully generated 2.2 kb *PHYA* GST sequences from *G. hirsutum*, *G. barbadense*, *G. herbaceum* and *G. raimondii* genomes (Abdurakhmonov et al. unpublished) that were aligned to design CAPS and dCAPS markers. Similarly, using PCR-walking approach, the first and second exons, part of the third exon, and first and second introns of *Gossypium HY5* were sequenced that include previously characterized *HY5* GST [35]. A 2.1 kb long *PHYB* GSTs of *Gossypium* species reported by Abdurakhmonov et al. [46] was used for development of *PHYB* specific markers. Sequencing, cloning and characterization of upstream and downstream regions via PCR-walking from the hinge region of targeted genes were conducted according to detailed methodology described by Abdurakhmonov et al. [46].

GSTs were aligned using Sequencher program ver. 4.1 (Gene Codes, USA) and GSP sites polymorphic between *G. hirsutum* and *G. barbadense* were determined. The polymorphic sites in cloned candidate genes were used to design gene-specific PCR-based CAPS markers. If identified polymorphism was recognized by commercially available restriction endonuclease CAPS markers were directly generated [4]; otherwise, a new SNP was artificially inserted near the GSP site to create a new restriction endonuclease recognition site using dCAPS Finder 2.0 [5, 9]. Marker primers were synthesized by Integrated DNA technologies Inc., (Iowa, USA) and used for genotyping experiments (Table 1).

### CAPS and dCAPS marker genotyping

For genotyping, the PCR amplifications were performed in a 10 μl reaction mixture containing 1 μl 10 × PCR buffer with MgCl_2_, 0.5 μl 25 mM of a dATP, dGTP, dTTP, and dCTP mix, 0.5 μl 25 ng/ml of each reverse and forward primer, 1 μl 10 ng/μl template DNA, and 0.1 U Taq DNA polymerase. PCR amplification was performed on a GeneAmp 9700 thermal cycler using the program consisting of an initial denaturation at 95 °C for 5 min, followed by 40 cycles of: denaturation at 95 °C for 45 s., annealing at 55–68 °C (depending on primers) for 45 sec. and elongation at 72 °C for 2 min., and finished with a final elongation at 72 °C for 10 min.

PCR products were purified using a 26 % PEG (polyethylene glycol) solution (PEG 8000, 6.5 mM MgCl_2_, 0.6 M NaOAc - pH 6.0–7.0) and digested with commercial restriction enzymes recognizing CAPS and dCAPS sites. Restriction analysis of each sample was performed in 10 μl of reaction mixture containing 1 μl 10 × restriction enzyme buffer, 2 μl purified PCR product, 0.2 Unit restriction enzyme and 6.5 μl sterile water. The digested products were electrophoresed on 3.5 % high-resolution agarose (HiRes Agarose) gel in 0.5 × TBE buffer, with a mode voltage of 5.3 V/cm. After electrophoresis, gels were stained with ethidium bromide (EtBr) solution for 5–10 min and photo-documented using Gel Imaging Documentation System (Alphaimager 2200, Alpha Innotech, USA) with exposure under the UV light.

### Construction of linkage maps and QTL analysis

To incorporate phytochrome and *HY5*-specific CAPS and dCAPS markers into the tetraploid cotton linkage map, we genotyped these markers in the bi-parental progenies of 186 RILs, which were developed from an interspecific cross between ‘TM-1’ and ‘Pima 3-79’ [48, 49]. The genetic linkage relationships with the reference genetic maps were constructed from the genotypic data of markers in RILs, using the program JoinMap version 3.0 [58]. Assignment of linkage groups to the respective chromosomes was based on the reference genetic maps of Yu et al. [48, 49]. For a graphical representation of QTL maps and linkage groups, the program Map Chart version 2.2 [58] was used. Previously mapped QTL information on specific linkage groups [48, 49] were also placed into CAPS and dCAPS marker-incorporated linkage groups to predict and interpret genetic association of targeted regions of cotton genome.

### Deletion analysis using chromosome substitution lines

The CS-B chromosome substitution lines were used for verification of chromosomal localization of CAPS and dCAPS markers. Each individual CS-B line is composed of *G. hirsutum* cv. ‘TM-1’ with a single chromosome or chromosome segment substituted from *G. barbadense* cv. ‘Pima 3–79’ [52]. In addition, an individual monosomic or monotelodisomic F_1_ stocks that lack a chromosome or one arm of a chromosome from the recipient, ‘TM-1’, and have the homologous chromosome or chromosome arm from the donor ‘Pima 3-79’ line was used. Amplified products of CAPS and dCAPS markers were assigned to the substituted chromosomes based on GSP polymorphisms and deletion method, in which the cytogenetic stock exhibited a hemizygous-banding pattern, with the ‘TM-1’ band is missing, in such cases, it could be considered that the locus was situated on that missing or the substituted chromosome or chromosome arm of the aneuploid or CS-B line. DNAs from *G. hirsutum* cv. ‘TM-1’, *G. barbadense* cv. ‘Pima 3–79’, and monotelodisomic and monosomic substitution lines (BC_0_F_1_) for different chromosomes and chromosome arms of *G. barbadense* were used to identify the chromosomal location of CAPS and dCAPS markers following the deletion analysis strategy used previously [59, 60]. The DNAs from individual aneuploid substitution lines were provided by Dr. D.M. Stelly at Texas A&M University, College Station, Texas [52].

## Additional file

Additional file 1: Figure S1a. The examples of PHYBdCAPs-2 markers segregating among TM-1 × 3–79 RIL lines. (M) – Molecular-weight size marker of 25-bp ladder, ‘TM-1’ and ‘Pima 3–79’ – parents, F_1_ – first-generation hybrid, 13–51 – RIL individuals. Note: the 144-bp band is more intensive in *G. hirsutum* and the 180-bp band is more intensive in *G. barbadense* genotypes, while 36-bp band is not visible to detect. Heterozygots show two intensive bands of 180- and 144-bp, respectively. Primer information for PHYBdCAPs-2: F-5’GAAGATCATAAAAAGGCTATATACGTGGTGGTTA3’; R-5’CAAAGGATTGGGACTATGAACAATGG3’; **Figure S1b.** Genetic linkage maps with integration of PHYBdCAPs and PHYBdCAPs-2 corresponding to chromosome 10 of the A-sub-genome [48, 49]. QTL designations on the map are follows as Ns - nep size; Nn - number of neps; UQL - upper quartile of fiber length by weight; SFC - short fiber content by weight g; ALFw - average length of all fiber by weight; 5.0 L - fiber span length; 2.5 L - fiber span length; VFM – visible foreign matter in percentage; FTX - fiber fineness; IFC - immature fiber content by weight g; MR - maturity ratio; MT - mean tenacity; and ME - mean elongation. (DOCX 820 kb)

## Abbreviations

‘TM-1’: ‘Texas Marker-1’
ALFw: Average length of all fibers by weight (g)
CAPS: Cleaved amplified polymorphisms
CS-B: Chromosome substitution-backcrossed line
CTAB: Cetyltrimethylammonium bromide
dCAPS: Derived-CAPS
FE: Fiber elongation
FTX: Fiber fineness
FU: Fiber uniformity
GSP: Genome-specific polymorphism
GST: Genome sequence tag
IFC: Immature fiber content
LY: Lint yield
MAS: Marker-assisted selection
MC: Micronaire
MT: Mean tenacity
NFB: Node of first fruiting branch
QTL: Quantitative trait loci
SFC: Short fiber content by weight (g)
SNAP: Single nucleotide-amplified polymorphisms
SNP: Single nucleotide polymorphism
SSCP: Single stranded confirmation polymorphism

## References

[1] Rafalski A (2002). Applications of single nucleotide polymorphisms in crop genetics. Curr Opin Plant Biol.

[2] Gupta PK, Roy JK, Prasad M (2001). Single nucleotide polymorphisms: a new paradigm for molecular marker technology and DNA polymorphism detection with emphasis on their use in plants. Curr Sci.

[3] Abdurakhmonov IY, Abdukarimov A (2008). Application of association mapping to understanding the genetic diversity of plant germplasm resources. Int J Plant Genomics.

[4] Konieczny A, Ausubel FM (1993). A procedure for mapping Arabidopsis mutations using co-dominant ecotype-specific PCR-based markers. Plant J.

[5] Neff MM, Neff JD, Chory J (1998). Pepper AE dCAPS, a simple technique for the genetic analysis of single nucleotide polymorphisms: experimental applications in *Arabidopsis thaliana* genetics. Plant J.

[6] Lee GA, Koh HJ, Chung HK, Dixit A, Chung JW, Ma KH, Lee SY, Lee JR, Lee GS, Gwag JG, Kim TS, Park YJ (2009). Development of SNP-based CAPS and dCAPS markers in eight different genes involved in starch biosynthesis in rice. Mol Breeding.

[7] Logan-Young CJ, Yu JZ, Verma SK, Percy RG, Pepper AE (2015). SNP discovery in complex allotetraploid genomes (*Gossypium* spp., Malvaceae) Using Genotyping by Sequencing. Appl Plant Sci.

[8] Michaels SD, Amasino RM (1998). A robust method for detecting single nucleotide changes as polymorphic markers by PCR. Plant J.

[9] Neff MM, Turk E, Kalishman M (2002). Web-based primer design for single nucleotide polymorphism analysis. Trends Genet.

[10] Akhunov ED, Akhunova AR, Linkiewicz AM, Dubcovsky J, Hummel D, Lazo G (2003). Synteny perturbations between wheat homoeologous chromosomes caused by locus duplications and deletions correlate with recombination rates. Proc Natl Acad Sci U S A.

[11] Blake NK, Sherman JD, Dvořák J, Talbert LE (2004). Genome-specific primer sets for starch biosynthesis genes in wheat. Theor Appl Genet.

[12] An C, Saha S, Jenkins JN, Scheffler BE, Wilkins TA, Stelly DM (2007). Transcriptome profiling, sequence characterization, and SNP-based chromosomal assignment of the EXPANSIN genes in cotton. Mol Genet Genomics.

[13] Buyyarapu R, Kantety RV, Yu JZ, Saha S, Sharma GC (2011). Development of new candidate gene and EST-based molecular markers for *Gossypium* species. Int J Plant Genomics.

[14] Van Deynze A, Stoffel K, Lee M, Wilkins TA, Kozik A, Cantrell RG, John ZY, Russel JK, David MS (2009). Sampling nucleotide diversity in cotton. BMC Plant Biol.

[15] Byers RL, Harker DB, Yourstone SM, Maughan PJ, Udall JA (2012). Development and mapping of SNP assays in allotetraploid cotton. Theor Appl Genet.

[16] Kumpatla SP, Buyyarapu R, Abdurakhmonov IY, Mammadov JA, Abdurakhmonov IY (2012). Genomics-assisted plant breeding in the 21st century: technological advances and progress. Plant Breeding.

[17] Hulse-Kemp AM, Ashrafi H, Zheng X, Wang F, Hoegenauer KA, Maeda AB (2014). Development and bin mapping of gene-associated interspecific SNPs for cotton (*Gossypium hirsutum* L.) introgression breeding efforts. BMC Genom.

[18] Li X, Gao W, Guo H, Zhang X, Fang DD, Lin Z (2014). Development of EST-based SNP and InDel markers and their utilization in tetraploid cotton genetic mapping. BMC Genom.

[19] Hulse-Kemp AM, Lemm J, Plieske J, Ashrafi H, Buyyarapu R, Fang DD (2015). Development of a 63K SNP array for cotton and high-density mapping of intra- and inter-specific populations of *Gossypium* spp. G3 (Bethesda).

[20] Wang S, Chen J, Zhang W, Hu Y, Chang L, Fang L (2015). Sequence-based ultra-dense genetic and physical maps reveal structural variations of allopolyploid cotton genomes. Genome Biol.

[21] Wang Y, Ning Z, Hu Y, Chen J, Zhao R, Chen H (2015). Molecular mapping of restriction-site associated DNA markers in allotetraploid upland cotton. PLoS One.

[22] Thyssen GN, Fang DD, Turley RB, Florane C, Li P, Naoumkina M (2015). Mapping-by-sequencing of Ligon-lintless-1 (Li 1) reveals a cluster of neighboring genes with correlated expression in developing fibers of Upland cotton (*Gossypium hirsutum* L.). Theor Appl Genet.

[23] Islam MS, Zeng L, Thyssen GN, Delhom CD, Kim HJ, Li P (2016). Mapping by sequencing in cotton (*Gossypium hirsutum*) line MD52ne identified candidate genes for fiber strength and its related quality attributes. Theor Appl Genet.

[24] Feng X, Keim D, Wanjugi H, Coulibaly I, Fu Y, Schwarz J (2015). Development of molecular markers for genetic male sterility in *Gossypium hirsutum*. Mol Breed.

[25] Wang F, Stewart JM, Zhang J (2007). Molecular markers linked to the Rf(2) fertility restorer gene in cotton. Genome.

[26] Rong J, Abbey C, Bowers JE, Brubaker CL, Chang C, Chee PW (2004). A 3347-locus genetic recombination map of sequence-tagged sites reveals features of genome organization, transmission and evolution of cotton (*Gossypium*). Genetics.

[27] Chen F, Li B, Li G, Charron JB, Dai M, Shi X (2014). Arabidopsis Phytochrome A directly targets numerous promoters for individualized modulation of genes in a wide range of pathways. Plant Cell.

[28] Smith H (1994). Phytochrome transgenics; functional, ecological and biotechnological applications. Semin Cell Biol.

[29] Robson PRH, Smith H (1997). Fundamental and biotechnological applications of phytochrome transgenes. Plant Cell Environ.

[30] Robson PR, McCormac AC, Irvine AS, Smith H (1996). Genetic engineering of harvest index in tobacco through overexpression of a phytochrome gene. Nat Biotechnol.

[31] Thiele A, Herold M, Lenk I, Quail PH, Gatz C (1999). Heterologous expression of Arabidopsis phytochrome B in transgenic potato influences photosynthetic performance and tuber development. Plant Physiol.

[32] Rao AQ, Irfan M, Saleem Z, Nasir IA, Riazuddin S, Husnain T (2011). Overexpression of the phytochrome B gene from Arabidopsis thaliana increases plant growth and yield of cotton (*Gossypium hirsutum*). J Zhejiang Univ Sci B.

[33] Fankhauser C, Chory J (1997). Light control of plant development. Annu Rev Cell Dev Biol.

[34] Kasperbauer MJ (2000). Cotton fiber length is affected by far-red light impinging on developing bolls. Crop Sci.

[35] Abdurakhmonov IY (2001). Molecular cloning and characterization of genomic sequence tags (GSTS) from the PHYA, PHYB, and HY5 gene families of cotton (Gossypium species).

[36] Abdurakhmonov IY, Buriev ZT, Saha S, Jenkins JN, Abdukarimov A, and Pepper AE. Cotton PHYA1 RNAi Improves fibre Quality, Root Elongation, Flowering, Maturity and Yield Potential in *Gossypium hirsutum* L. U S A. Patent Application. 2013. http://www.google.com/patents/US20130227723. Accessed 21 Aug 2016.

[37] Abdurakhmonov IY, Buriev ZT, Saha S, Jenkins JN, Abdukarimov A, Pepper AE (2014). Phytochrome RNAi enhances major fibre quality and agronomic traits of the cotton *Gossypium hirsutum* L. Nat Commun.

[38] Datta S, Hettiarachchi C, Johansson H, Holm M (2007). SALT TOLERANCE HOMOLOG2, a B-box protein in Arabidopsis that activates transcription and positively regulates light-mediated development. Plant Cell.

[39] Datta S, Johansson H, Hettiarachchi C, Irigoyen ML, Desai M, Rubio V, Holm M (2008). LZF1/SALT TOLERANCE HOMOLOG3, an Arabidopsis B-box protein involved in light-dependent development and gene expression, undergoes COP1-mediated ubiquitination. Plant Cell.

[40] Jonassen EM, Lea US, Lillo C (2008). HY5 and HYH are positive regulators of nitrate reductase in seedlings and rosette stage plants. Planta.

[41] Lillo C (2008). Signalling cascades integrating light-enhanced nitrate metabolism. Biochem J.

[42] Beck EH, Fettig S, Knake C, Hartig K, Bhattarai T (2007). Specific and unspecific responses of plants to cold and drought stress. J Biosci.

[43] Franklin KA, Whitelam GC (2007). Light-quality regulation of freezing tolerance in Arabidopsis thaliana. Nat Genet.

[44] Kim HJ, Kim YK, Park JY, Kim J (2002). Light signalling mediated by phytochrome plays an important role in cold-induced gene expression through the C-repeat/dehydration responsive element (C/DRE) in *Arabidopsis thaliana*. Plant J.

[45] Xie XZ, Xue YJ, Zhou JJ, Zhang B, Chang H, Takano M (2011). Phytochromes regulate SA and JA signaling pathways in rice and are required for developmentally controlled resistance to Magnaporthe grisea. Mol Plant.

[46] Abdurakhmonov IY, Buriev ZT, Logan-Young CJ, Abdukarimov A, Pepper AE (2010). Duplication, divergence and persistence in the phytochrome photoreceptor gene family of cottons (*Gossypium* spp.). BMC Plant Biol.

[47] Abdurakhmonov IY, Ayubov MS, Ubaydullaeva KA, Buriev ZT, Shermatov SE, Ruziboev HS (2016). RNA interference for functional genomics and improvement of cotton (*gossypium* spp.). front. Plant Sci.

[48] Yu JZ, Kohel RJ, Fang DD, Cho J, Van Deynze A, Ulloa M (2012). A high-density simple sequence repeat and single nucleotide polymorphism genetic map of the tetraploid cotton genome. G3 (Bethesda).

[49] Yu JZ, Ulloa M, Hoffman SM, Kohel RJ, Pepper AE, Fang DD (2014). Mapping genomic loci for cotton plant architecture, yield components, and fiber properties in an interspecific (*Gossypium hirsutum* L. × *G. barbadense* L.) RIL population. Mol Genet Genomics.

[50] Saha S, Raska DA, Stelly DM (2006). Upland Cotton (*Gossypium hirsutum* L.) x Hawaiian Cotton (*G. tomentosum* Nutt. Ex. Seem.) F1 Hybrid Hypoaneuploid Chromosome Substitution Series. J Cotton Sci.

[51] Saha S, Stelly DM, Raska DA, Wu J, Jenkins JN, McCarty JC, Abdurakhmonov IY (2012). Chromosome substitution lines: concept, development and utilization in the genetic improvement of Upland cotton. Plant Breeding.

[52] Stelly D, Saha S, Raska D, Jenkins J, McCarty J, Gutierrez O (2005). Registration of 17 Upland (*Gossypium hirsutum*) germplasm lines disomic for different G. barbadense chromosome or arm substitutions. Crop Sci.

[53] Yu J, Yu S, Gore M, Wu M, Zhai H, Li X (2013). Identification of quantitative trait loci across interspecific F2, F2:3 and testcross populations for agronomic and fiber traits in tetraploid cotton. Euphytica.

[54] Guo Y, McCarty JC, Jenkins JN, An C, Saha S (2009). Genetic detection of node of first fruiting branch in crosses of a cultivar with two exotic accessions of upland cotton. Euphytica.

[55] Said JI, Lin Z, Zhang X, Song M, Zhang J (2013). A comprehensive meta QTL analysis for fiber quality, yield, yield related and morphological traits, drought tolerance, and disease resistance in tetraploid cotton. BMC Genom.

[56] Wang B, Nie Y, Lin Z, Zhang X, Liu J, Bai J (2012). Molecular diversity, genomic constitution, and QTL mapping of fiber quality by mapped SSRs in introgression lines derived from Gossypium hirsutum x G. darwinii Watt. Theor Appl Genet.

[57] Dellaporta SL, Wood J, Hicks JB (1983). A plant DNA mini- preparation: version II. Plant Mol Biol Rep.

[58] Van Ooijen JW, Boer MP, Jansen RC, Maliepaard C (2002). MapQTL 4.0, software for the calculation of QTL positions of genetic maps.

[59] Abdurakhmonov IY, Buriev ZT, Saha S, Pepper AE, Musaev JA, Almatov A (2007). Microsatellite markers associated with lint percentage trait in cotton, *Gossypium hirsutum*. Euphytica.

[60] Saha S, Stelly DM, Makamov A, Ayubov MS, Raska D, Gutiérrez OA (2015). Molecular confirmation of *Gossypium hirsutum* chromosome substitution lines. Euphytica.

