# Supplementary material for: Development, genetic mapping and QTL association of cotton PHYA, PHYB, and HY5-specific CAPS and dCAPS markers
Source: BMC Genet. 2016 Oct 24;17:141. doi: 10.1186/s12863-016-0448-4 (PMC5078887; doi:10.1186/s12863-016-0448-4)
Supplement: Additional file 1: Figure S1a. — The examples of PHYBdCAPs-2 markers segregating among TM-1 × 3–79 RIL lines. (M) – Molecular-weight size marker of 25-bp ladder, ‘TM-1’ and ‘Pima 3–79’ – parents, F1 – first-generation hybrid, 13–51 – RIL individuals. Note: the 144-bp band is more intensive in G. hirsutum and the 180-bp band is more intensive in G. barbadense genotypes, while 36-bp band is not visible to detect. Heterozygots show two intensive bands of 180- and 144-bp, respectively. Primer information for PHYBdCAPs-2: F-5’GAAGATCATAAAAAGGCTATATACGTGGTGGTTA3’; R-5’CAAAGGATTGGGACTATGAACAATGG3’; Figure S1b. Genetic linkage maps with integration of PHYBdCAPs and PHYBdCAPs-2 corresponding to chromosome 10 of the A-sub-genome [48, 49]. QTL designations on the map are follows as Ns - nep size; Nn - number of neps; UQL - upper quartile of fiber length by weight; SFC - short fiber content by weight g; ALFw - average length of all fiber by weight; 5.0 L - fiber span length; 2.5 L - fiber span length; VFM – visible foreign matter in percentage; FTX - fiber fineness; IFC - immature fiber content by weight g; MR - maturity ratio; MT - mean tenacity; and ME - mean elongation. (DOCX 820 kb) [file 12863_2016_448_MOESM1_ESM.docx]

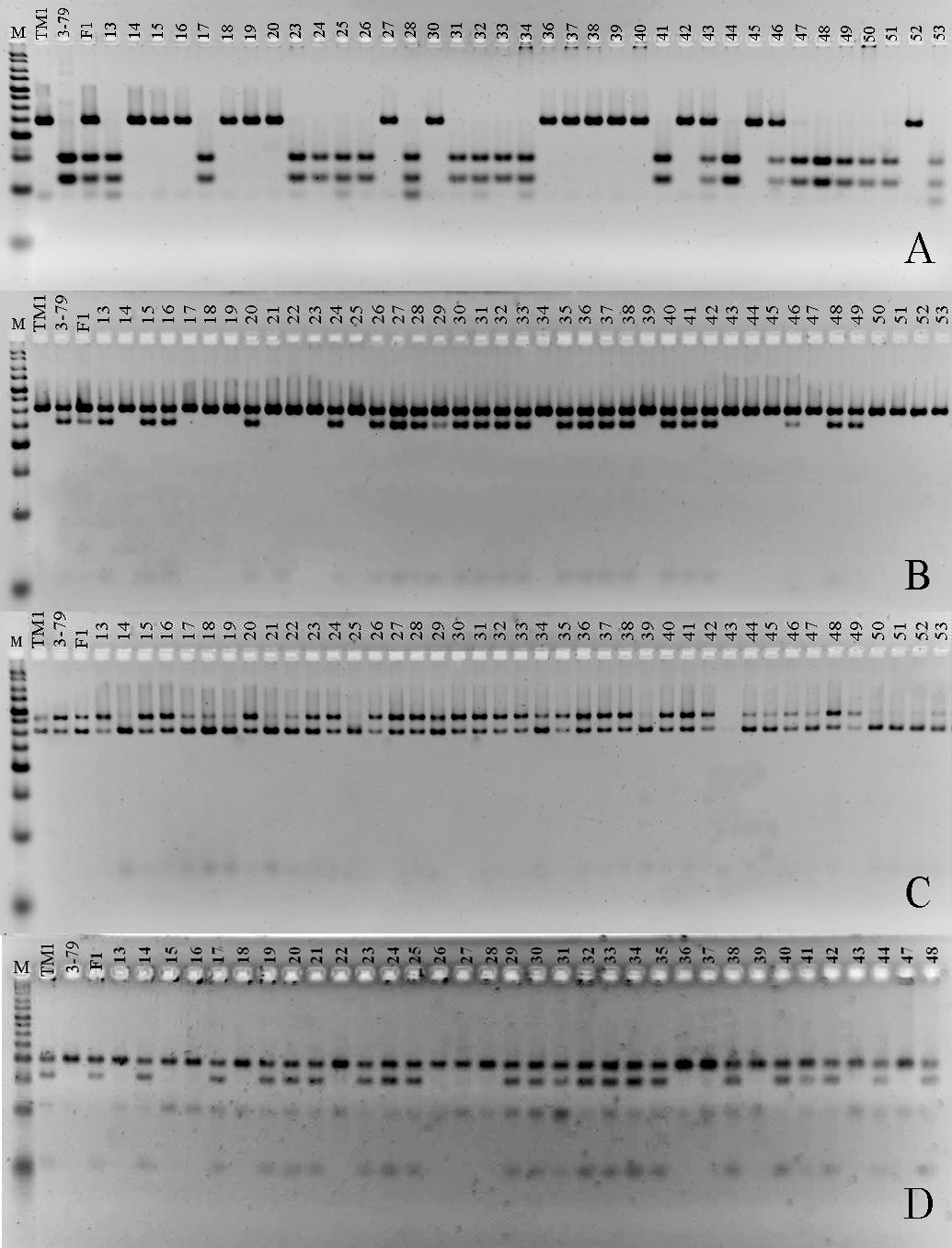


**Additional File Figure 1a.** The examples of PHYBdCAPs-2 markers segregating among TM-1 x 3-79 RIL lines. (М) – Molecular-weight size marker of 25-bp ladder, ‘ТМ-1’ and ‘Pima 3-79’ – parents, F_1_ – first-generation hybrid, 13-51 – RIL individuals. Note: the 144-bp band is more intensive in *G. hirsutum* and the 180-bp band is more intensive in *G. barbadense* genotypes, while 36-bp band is not visible to detect. Heterozygots show two intensive bands of 180- and 144-bp, respectively. Primer information for PHYBdCAPs-2: F-5’GAAGATCATAAAAAGGCTATATACGTGGTGGTTA3’; R-5’CAAAGGATTGGGACTATGAACAATGG3’.

**Additional File Figure 1b.** Genetic linkage maps with integration of PHYBdCAPs
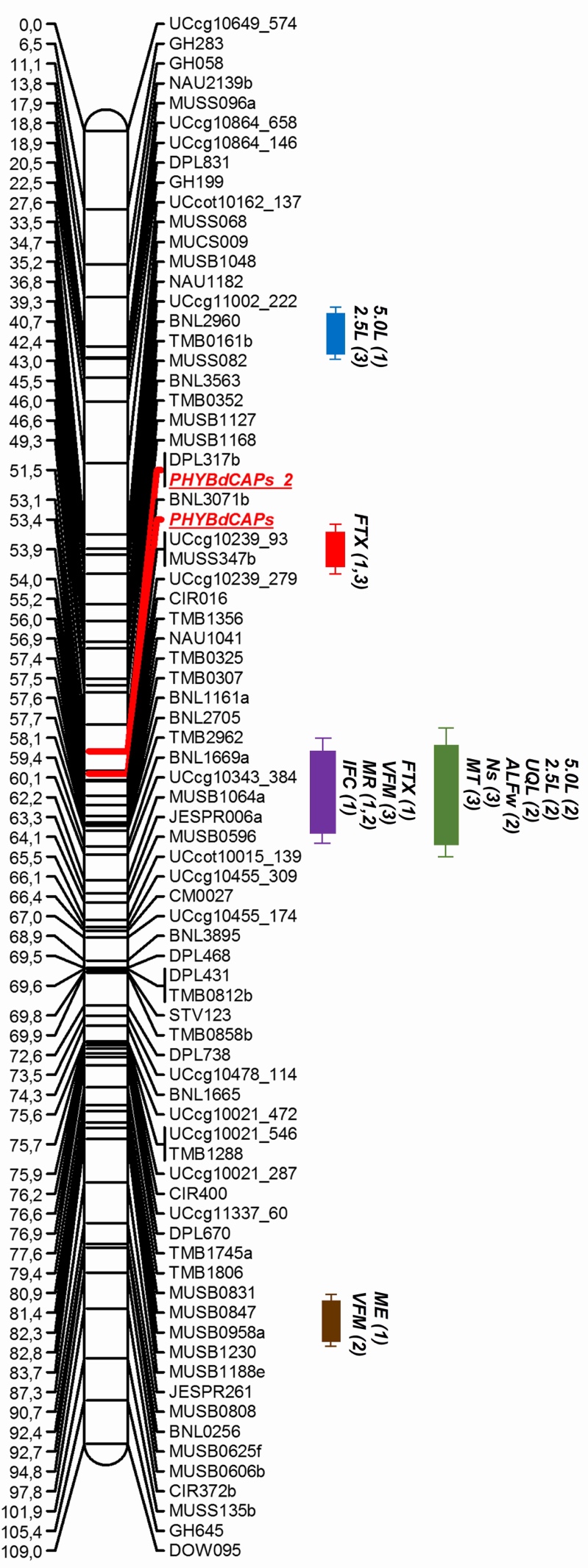
and PHYBdCAPs-2 corresponding to chromosome 10 of the A-sub-genome [48, 49]. QTL designations on the map are follows as **Ns** - nep size; **Nn** - number of neps; **UQL** - upper quartile of fiber length by weight; **SFC** - short fiber content by weight g; **ALFw** - average length of all fiber by weight; **5.0L** - fiber span length; **2.5L** - fiber span length; **VFM** – visible foreign matter in percentage; **FTX** - fiber fineness; **IFC** - immature fiber content by weight g; **MR** - maturity ratio; **MT** - mean tenacity; and **ME** - mean elongation.
